# Psychological and somatosensory correlates of temporomandibular disorder: anxiety, somatosensory amplification, and coping strategies—a biopsychosocial perspective

**DOI:** 10.22514/jofph.2026.014

**Published:** 2026-05-12

**Authors:** Zekeriya Temircan

**Affiliations:** ^1^Department of Psychology, Cappadocia University, 50420 Nevşehir, Türkiye

**Keywords:** Temporomandibular disorder, Anxiety, Somatosensory amplification, Coping strategies, Biopsychosocial model, Pain

## Abstract

**Background**: Temporomandibular disorders (TMDs) are multifactorial conditions influenced by physical, psychological, and behavioral factors. Emotional distress, altered pain perception, and maladaptive coping strategies contribute to TMD pathology. This study examined psychological and somatosensory correlates of TMDs, focusing on anxiety, somatosensory amplification, and coping styles. **Methods**: This cross-sectional study included 108 patients with TMDs (76 females, 32 males; mean age 33.98 ± 7.20 years) and 112 healthy controls (58 females, 54 males; mean age 34.25 ± 6.85 years). Anxiety was assessed using the Hospital Anxiety and Depression Scale–Anxiety subscale (HADS-A), somatosensory amplification with the Somatosensory Amplification Scale (SSAS), and coping strategies with the Coping Orientation to Problems Experienced (COPE) Inventory. Pain intensity and symptom duration were measured using the Visual Analog Scale (VAS). Group differences were analyzed using independent-samples *t*-tests and chi-square tests. Pearson correlation and multivariate regression analyses were conducted within the TMD group to identify predictors of somatosensory amplification. Ethical approval was obtained from Cappadocia University. **Results**: Patients with TMDs showed significantly higher anxiety (HADS-A: 11.42 ± 3.85 *vs.* 6.18 ± 2.90, *p* < 0.001) and somatosensory amplification (SSAS: 31.75 ± 7.92 *vs.* 20.84 ± 8.33, *p* < 0.001) compared with controls. The TMD group reported greater use of emotion-focused and maladaptive coping strategies (*p* ≤ 0.01), while problem-focused coping did not differ. Pain intensity (VAS: 6.48 ± 1.72) and symptom duration (4.62 ± 2.74 years) were positively correlated with anxiety, somatosensory amplification, and maladaptive coping (all *p* < 0.001). Regression analyses identified pain intensity and anxiety as significant predictors of somatosensory amplification, with female gender and older age also contributing. **Conclusions**: These findings support a biopsychosocial model of TMDs, highlighting interactions among pain, emotional distress, somatosensory amplification, and coping strategies. Integrative treatments addressing both physical and psychological factors may improve outcomes.

## 1. Introduction

Temporomandibular disorders (TMDs) constitute a heterogeneous group of musculoskeletal conditions affecting the temporomandibular joint (TMJ), masticatory muscles, and associated structures [1]. Owing to their multifactorial and complex nature, the diagnosis and management of TMDs have historically posed clinical challenges [2]. Although substantial research has highlighted the influence of psychological factors on TMDs [3, 4], inconsistencies in measurement tools and limitations in diagnostic criteria have contributed to ambiguity in understanding their precise role. Accumulating evidence indicates that, beyond structural pathology, behavioral and psychological components considerably shape the chronic pain experience and overall quality of life in individuals with TMDs [5].

Even when no clear organic etiology is identified, many patients with TMDs present with psychiatric or psychosomatic symptoms, underscoring the role of psychological factors in symptom development. Personality traits [6], emotional distress [7], depression [8], and somatization [9] are commonly reported in individuals with TMDs and require careful evaluation by mental health professionals [10]. The longstanding interest in the association between TMDs, anxiety, and depression reflects the ongoing uncertainty surrounding TMDs etiology and the recognized influence of psychological, behavioral, and environmental factors [11]. Psychosocial variables are now widely considered predisposing or perpetuating contributors to TMDs, with anxiety, depression, and emotional distress exerting significant effects on TMJ function and pain experiences [12, 13]. Pain particularly myofascial pain remains the most commonly reported symptom and often the primary reason patients seek treatment [14]. Importantly, this pain may originate from musculoskeletal dysfunction or may represent a somato-psychological or psychiatric phenomenon [15]. Supporting this view, studies have demonstrated significant associations between myofascial pain, anxiety, depression, and somatization [16].

Somatosensory disturbances, such as increased pain sensitivity, are common features of chronic pain and have been frequently reported in TMDs [17]. Although impaired endogenous pain modulation has been described in the literature, its assessment requires specialized experimental paradigms and cannot be routinely determined in standard clinical evaluations [18]. Importantly, these disturbances do not imply a purely psychiatric origin of pain but rather reflect the interaction between peripheral nociceptive mechanisms and central biopsychosocial processes. Accordingly, contemporary research widely supports the application of a biopsychosocial model in the evaluation of TMDs, emphasizing the need to consider both somatic and psychological components in clinical assessment [19]. Psychological factors such as psychosocial stress, somatic symptom burden, and affective distress have been shown to predict both the onset and persistence of TMDs symptoms [20]. Moreover, anxiety and depression are consistently linked to various clinical manifestations of TMDs [21]. In contrast, some studies conducted in pain-free populations have reported weaker associations between trait anxiety and oral parafunctional behaviors, suggesting that the impact of psychological factors may become more pronounced in the presence of chronic pain conditions such as TMDs [22].

Within a biopsychosocial perspective, coping strategies constitute an important psychological process shaping pain perception and functional adjustment in chronic pain conditions. Coping involves cognitive and behavioral responses used to manage pain-related stressors. Non-functional coping refers to maladaptive or avoidant strategies (*e.g.*, denial, behavioral disengagement, substance use) that do not effectively reduce stress and may exacerbate pain-related distress, whereas emotion-focused coping aims to regulate emotional responses to stress without directly addressing the stressor. Evidence from chronic pain research indicates that greater use of emotion-focused or non-functional coping (maladaptive coping) is associated with higher pain intensity and emotional distress, whereas problem-focused coping is linked to more adaptive outcomes [23]. In TMDs, maladaptive coping may facilitate symptom persistence by amplifying pain-related attention and somatic sensitivity, underscoring its relevance for clinical assessment and intervention [24].

Collectively, evidence points to a complex interplay between somatosensory processes, emotional distress, and functional impairment in TMDs. Symptoms of anxiety, depression, and somatization appear to be elevated among patients with TMDs compared to healthy controls and are often associated with greater pain intensity, jaw functional limitations, and maladaptive behavioral patterns [25]. These findings underscore the importance of investigating both psychological correlates such as anxiety, somatosensory amplification (SSAS), and coping strategies and somatosensory alterations to better understand the biopsychosocial mechanisms underlying TMDs.

Importantly, anxiety, somatosensory amplification, and coping strategies represent complementary yet distinct components of the biopsychosocial pain model. Anxiety reflects emotional distress and affective vulnerability, somatosensory amplification captures heightened perceptual sensitivity to bodily sensations, and coping strategies reflect behavioral and cognitive responses to pain-related stress. Together, these constructs allow for a multidimensional evaluation of psychological distress, perceptual processing, and adaptive or maladaptive behavioral regulation in TMDs. Examining these variables concurrently may therefore provide a more integrative understanding of the mechanisms contributing to pain persistence and functional impairment in individuals with TMDs.

Based on the above literature, the present study aimed to investigate the psychological and somatosensory correlates of TMDs, focusing specifically on anxiety, somatosensory amplification, and coping strategies. This study hypothesized that individuals with TMDs would exhibit higher levels of emotional distress and somatosensory amplification compared with healthy controls, and that maladaptive coping strategies would be associated with greater pain intensity and somatic sensitivity. This research seeks to clarify the interrelationships among these factors within a biopsychosocial framework and provide guidance for targeted assessment and intervention strategies.

## 2. Materials and methods

### 2.1 Subjects

The study employed a cross-sectional design and included 108 adult patients diagnosed with TMDs according to the Diagnostic Criteria for Temporomandibular Disorders (DC/TMD) [26], recruited from Kapadokya Hospital between June 2024 and December 2024. Recruitment was entirely voluntary. Reflecting the heterogeneous nature of TMDs, the patient group included 62 individuals (57.4%) with myogenous TMD, 21 (19.4%) with arthrogenous TMD, and 25 (23.1%) with combined (myogenous + arthrogenous) TMD.

Control participants were recruited through public announcements and hospital notice boards. To enhance group comparability, the control group was selected to be broadly comparable to the TMD group in terms of key demographic characteristics, including age and sex. No formal individual matching procedure was applied; however, demographic variables were examined and did not differ significantly between groups. To ensure that control participants were free of TMD and other pain symptoms, all individuals underwent a structured clinical screening, including a DC/TMD examination and self-report questionnaires about current or chronic pain conditions. Participants reporting any pain or showing clinical signs of TMD were excluded. Written informed consent was obtained from all participants, and both sexes were represented to maintain a balanced and representative sample. The ethical approval was obtained from Cappadocia University ethics committee.

A *post-hoc* power analysis using G*Power 3.1 (Heinrich Heine University Düsseldorf, Düsseldorf, NRW, Germany) for multiple regression (5 predictors, medium effect size *f*^2^ = 0.15, α = 0.05) indicated that a minimum of 92 participants would be required to achieve 80% power. With a total sample of 220 participants, the study is sufficiently powered to detect medium effect sizes in regression and correlation analyses.

Within the biopsychosocial framework adopted in the present study, the biological domain was operationalized through clinical pain characteristics, including pain intensity and TMD diagnosis. The psychological domain was represented by measures of emotional distress (anxiety), SSAS, and coping strategies, reflecting affective, perceptual, and cognitive–behavioral processes related to pain. The social and behavioral domain was indirectly captured through coping patterns, which reflect learned behavioral responses and adaptive or maladaptive strategies for managing pain-related stress within daily life contexts. This operationalization allows for an integrated assessment of the biological, psychological, and social dimensions contributing to TMDs.

### 2.2 Inclusion criteria

Study Groups: TMDs Group: Adult patients diagnosed with TMDs according to DC/TMD diagnostic criteria and voluntarily recruited from Kapadokya Hospital between June 2024 and December 2024.

Healthy Control Group: Volunteers from the local community recruited through public announcements and hospital notice boards. Both male and female participants were included to ensure representativeness.

### 2.3 Exclusion criteria

Participants were excluded if they had a history of prior TMDs treatment, systemic diseases affecting the temporomandibular joints (*e.g.*, rheumatoid arthritis, tumors, or trauma), neurological or psychiatric disorders, current use of medications influencing TMDs symptoms (*e.g.*, anxiolytics, antidepressants), or inability to read, understand, or complete the study questionnaires.

### 2.4 Questionnaires

#### 2.4.1 A sociodemographic form

This form was used to collect information on age, gender, duration of TMDs, and pain intensity assessed with the VAS. The severity of pain was evaluated using the Visual Analog Scale (VAS), a widely validated and reliable tool for assessing musculoskeletal pain. Participants indicated the intensity of their pain on a 10-cm line, where 0 represented no pain and 10 indicated unbearable pain. Participants were asked to report their current pain intensity at the time of assessment, as well as their average and worst pain over the past week, to capture both immediate and recent pain experiences. Although current, average, and worst pain intensity over the past week were assessed using the Visual Analog Scale (VAS), only the average pain score was used in the statistical analyses. Average pain was selected as it provides a more stable and representative estimate of participants’ typical pain experience over time, reducing the influence of transient fluctuations associated with momentary or peak pain intensity. This approach is commonly used in chronic pain research to enhance the reliability and interpretability of pain-related associations.

#### 2.4.2 Hospital anxiety and depression scale (HADS)

The Hospital Anxiety and Depression Scale (HADS) is a self-report measure developed by Zigmond and Snaith [27] to evaluate the level and severity of anxiety and depressive symptoms. It includes 14 items rated on a four-point Likert scale. The odd-numbered items form the anxiety subscale (HADS-A), while the even-numbered items comprise the depression subscale (HADS-D). The Turkish adaptation, validated by Aydemir *et al*. [28] confirmed that the instrument is suitable for identifying anxiety and depression in individuals with medical conditions. For the Turkish version, the recommended cut-off scores are ≥11 for the HADS anxiety subscale (HADS-A) and ≥8 for the HADS depression subscale (HADS-D), with scores equal to or above these thresholds indicating elevated risk. Both subscales provide total scores ranging from 0 to 21. In the present study, internal consistency was assessed using Cronbach’s alpha coefficients. The Hospital Anxiety and Depression Scale (HADS) demonstrated good internal consistency, with a Cronbach’s alpha of 0.82 for the anxiety subscale and 0.76 for the depression subscale.

#### 2.4.3 Somatosensory amplification scale (SSAS)

The SSAS is a widely used instrument designed to assess individuals’ heightened sensitivity to benign or routine bodily sensations. Developed by Barsky *et al*. [29] it includes 10 items, each rated on a 5-point Likert scale, with higher total scores indicating greater SSAS. The scale was adapted into Turkish by Güleç and Sayar [30] who demonstrated its adequate psychometric properties. The Turkish version showed acceptable internal consistency, with Cronbach’s alpha coefficients reported between 0.62 and 0.76, and a test–retest reliability of 0.73, indicating stability over time. The validated Turkish version of the SSAS was used. Scores ≥30 are generally interpreted as indicating high SSAS. In the present study, internal consistency was assessed using Cronbach’s alpha coefficients. The SSAS demonstrated good internal consistency, with a Cronbach’s alpha of 0.92.

#### 2.4.4 COPE assessment scale

The COPE Inventory was originally developed by Carver *et al*. [31] to assess individuals’ coping responses when faced with stressful or anxiety-provoking situations. The Turkish adaptation, including validity and reliability analyses, was conducted by Ağargün *et al*. [32]. The instrument consists of 60 items organized into 15 subscales, each reflecting a distinct coping strategy used to manage stress. In accordance with the Turkish COPE classification, these subscales were grouped into problem-focused, emotion-focused, and non-functional coping strategies. Problem-focused coping refers to active strategies aimed at addressing or modifying the stressor itself (*e.g.*, planning, active coping). Emotion-focused coping involves strategies aimed at regulating emotional responses to stress without directly altering the stressor (*e.g.*, emotional support, acceptance). Non-functional coping encompasses maladaptive or avoidant strategies that may hinder effective stress management (*e.g.*, denial, behavioral disengagement, substance use). The reliability of the Turkish version of the COPE Inventory was evaluated using Cronbach’s alpha and correlation analyses. The scale demonstrated high internal consistency (Cronbach’s α = 0.79), and subscale scores were positively and significantly correlated with the total COPE score. Test–retest reliability was also reported to be high. In the present study, internal consistency was assessed using Cronbach’s alpha coefficients, and the COPE demonstrated good internal consistency (Cronbach’s α = 0.77).

### 2.5 Statistical analysis

All statistical analyses were performed using SPSS version 28.0 (IBM Corp., Armonk, NY, USA). The normality of continuous variables was assessed using the Shapiro-Wilk test and visual inspection of histograms and Quantile-Quantile (Q-Q) plots. As all variables met the assumptions of normality, parametric tests were applied throughout the analyses. Group differences between patients with TMDs and healthy controls were analyzed using independent-samples *t*-tests for continuous variables and chi-square tests for categorical variables. Pearson correlation coefficients were used to examine relationships between pain intensity (VAS), anxiety (HADS-A), somatosensory amplification (SSAS), and coping strategies. Subgroup analyses were conducted within the TMDs group to explore associations specific to patients. Multivariate linear regression analyses were performed to identify predictors of SSAS and coping strategies in the TMDs group. Independent variables included age, gender, pain intensity, anxiety, and emotion-focused coping. Standardized beta coefficients (β) and unstandardized coefficients (B) were reported to indicate the magnitude and direction of effects. Regression assumptions, including linearity, homoscedasticity, and independence of residuals, were checked. All tests were two-tailed, and *p*-values < 0.05 were considered statistically significant.

## 3. Results

As shown in Table 1, individuals with TMDs (n = 108) exhibited significantly higher anxiety levels (HADS-A: 11.42 ± 3.85) than healthy controls (n = 112; 6.18 ± 2.90; *p* < 0.001). Somatosensory amplification was also significantly elevated in the TMDs group (SSAS: 31.75 ± 7.92) compared with controls (20.84 ± 8.33; *p* < 0.001). With respect to coping strategies, participants with TMDs reported greater use of emotion-focused coping (26.84 ± 6.12 *vs.* 22.40 ± 5.78; *p* = 0.001) and non-functional coping (22.91 ± 5.33 *vs.* 18.66 ± 4.92; *p* < 0.001), whereas problem-focused coping did not differ significantly between groups (18.62 ± 4.45 *vs.* 20.14 ± 4.12; *p* = 0.072). Clinically, the TMDs group reported moderate-to-severe pain intensity (VAS: 6.48 ± 1.72) and a mean symptom duration of 4.62 ± 2.74 years, indicating the chronic nature of the disorder. Overall, these findings indicate that TMDs is characterized by elevated emotional distress, increased somatosensory amplification, and a greater reliance on maladaptive coping strategies, supporting a biopsychosocial conceptualization of the condition.

**Table 1.** Comparison of sociodemographic, psychobehavioral, and clinical variables between the TMDs group and healthy controls.

Variables		TMDs group (n = 108)	Healthy controls (n = 112)	Statistic	*p*
Sociodemographic variables					
	Gender (Female/Male)	76/32	58/54	χ^2^ = 5.214	0.022*
	Age (yr)	33.98 ± 7.20	34.25 ± 6.85	*t* = −0.192	0.848
Psychobehavioral variables					
	Anxiety (HADS-A)	11.42 ± 3.85	6.18 ± 2.90	*t* = 7.036	<0.001**
	SSAS	31.75 ± 7.92	20.84 ± 8.33	*t* = 6.812	<0.001**
	Problem-focused coping	18.62 ± 4.45	20.14 ± 4.12	*t* = −1.822	0.072
	Emotion-focused coping	26.84 ± 6.12	22.40 ± 5.78	*t* = 3.479	0.001*
	Non-functional coping	22.91 ± 5.33	18.66 ± 4.92	*t* = 3.899	<0.001**
Clinical variables (TMDs group only)					
	Pain Intensity (VAS)	6.48 ± 1.72	—	—	—
	Duration of TMD (yr)	4.62 ± 2.74	—	—	—

Student’s t-test or chi-square test applied as appropriate. *p < 0.05; **p < 0.001 compared with the healthy group. TMD: Temporomandibular Disorders; HADS-A: Hospital Anxiety and Depression Scale–Anxiety; SSAS: Somatosensory Amplification Scale; VAS: Visual Analog Scale.

As shown in Table 2, across all participants, pain intensity was significantly and positively correlated with anxiety (*r* = 0.46, *p* < 0.001), SSAS (*r* = 0.41, *p* < 0.001), and non-functional coping (*r* = 0.34, *p* < 0.001). In the TMDs patient subgroup, pain intensity was significantly correlated with anxiety (*r* = 0.58, *p* < 0.001), SSAS (*r* = 0.49, *p* < 0.001), and non-functional coping (*r* = 0.47, *p* < 0.001). Anxiety in the TMDs group was also positively correlated with SSAS (*r* = 0.61, *p* < 0.001) and non-functional coping (*r* = 0.55, *p* < 0.001).

**Table 2.** Correlation results for all participants and TMDs group.

		Anxiety	SSAS	Emotion-focused coping	Non-functional coping
All participants (n = 220)					
	Pain intensity (VAS)	*r* = 0.46, *p* < 0.001**	*r* = 0.41, *p* < 0.001**	*r* = 0.29, *p* = 0.002*	*r* = 0.34, *p* < 0.001**
	Anxiety	1	*r* = 0.52, *p* < 0.001**	*r* = 0.37, *p* < 0.001**	*r* = 0.48, *p* < 0.001**
	SSAS	—	1	*r* = 0.31, *p* = 0.001*	*r* = 0.44, *p* < 0.001**
	Emotion-focused coping	—	—	1	*r* = 0.42, *p* < 0.001**
	Non-functional coping	—	—	—	1
Patients with TMDs only (n = 108)					
	Pain intensity (VAS)	*r* = 0.58, *p* < 0.001**	*r* = 0.49, *p* < 0.001**	*r* = 0.33, *p* = 0.001*	*r* = 0.47, *p* < 0.001**
	Anxiety	1	*r* = 0.61, *p* < 0.001**	*r* = 0.42, *p* < 0.001**	*r* = 0.55, *p* < 0.001**
	SSAS	—	1	*r* = 0.36, *p* < 0.001**	*r* = 0.50, *p* < 0.001**
	Emotion-focused coping	—	—	1	*r* = 0.46, *p* < 0.001**
	Non-functional coping	—	—	—	1

*p < 0.05; **p < 0.001. TMDs: Temporomandibular Disorders group; SSAS: Somatosensory amplification; VAS: Visual Analog Scale.

Multivariate regression analysis was conducted to examine predictors of SSAS in patients with TMDs. The independent variables included pain intensity (VAS), anxiety (HADS-A), age, gender, and emotion-focused coping. The analysis showed that pain intensity was significantly associated with SSAS (B = 0.451, β = 0.392, *p* < 0.001), indicating a moderate-to-large relationship. Anxiety also emerged as a significant predictor (B = 0.367, β = 0.341, *p* < 0.001), demonstrating a moderate association with SSAS. Age showed a small but significant effect (B = 0.132, β = 0.175, *p* = 0.024), while female gender was associated with higher SSAS scores (B = 2.815, β = 0.238, *p* = 0.012), reflecting a small-to-moderate effect. Emotion-focused coping had a small significant effect on SSAS (B = 0.142, β = 0.138, *p* = 0.036). The regression model accounted for a substantial portion of the variance in SSAS among patients with TMDs, and the detailed results are presented in Table 3.

**Table 3.** Multivariate regression analysis predicting somatosensory amplification in patients with TMDs.

Independent variables	B	SE	Beta	*t*	*p*
Age	0.132	0.058	0.175	2.276	0.024*
Gender	2.815	1.104	0.238	2.550	0.012*
Pain intensity (VAS)	0.451	0.072	0.392	6.269	<0.001**
Anxiety (HADS-A)	0.367	0.061	0.341	6.016	<0.001**
Emotion-focused coping	0.142	0.067	0.138	2.119	0.036*
Constant	15.724	3.218	—	4.888	<0.001**

*p < 0.05; **p < 0.001. TMD: Temporomandibular Disorders group; VAS: Visual Analog Scale; HADS-A: Hospital Anxiety and Depression Scale–Anxiety; B: Unstandardized regression coefficient; SE: Standard Error.

Regression analyses were performed to examine predictors of SSAS and coping strategies in the TMDs group. The results indicated that pain intensity was a significant predictor of SSAS (β = 0.487, *p* < 0.001), demonstrating a moderate-to-large effect. Anxiety was also a significant predictor of SSAS (β = 0.361, *p* < 0.001), showing a moderate effect size. In addition, anxiety significantly predicted coping strategies (β = 0.398, *p* = 0.002), indicating a moderate effect. These analyses highlight that both pain intensity and anxiety are associated with somatosensory amplification, and that anxiety is additionally related to coping strategies in patients with TMDs (Fig. 1).

**Fig. 1.** Path diagram showing the associations between pain intensity, anxiety, coping strategies, and somatosensory amplification (SSAS) in patients with temporomandibular disorders (TMDs).

All relevant coefficients, effect sizes, and statistical significance values are presented in Table 4.

**Table 4.** Effects of pain intensity and anxiety on somatosensory amplification and coping strategies in TMDs group.

Independent variable		Model 1 β	*R*	*R* ^2^	*F*	*p*
(A) Effects of Pain Intensity and Anxiety on SSAS						
	Pain Intensity (VAS)	0.487	0.487	0.237	7.921	<0.001**
	Anxiety (HADS-A)	0.361	0.524	0.274	9.102	<0.001**
(B) Effects of Anxiety on Coping Strategies						
	Anxiety (HADS-A)	0.398	0.398	0.158	5.322	0.002*

*p < 0.05; **p < 0.001. TMD: Temporomandibular Disorders group; SSAS: Somatosensory amplification; VAS: Visual Analog Scale; HADS-A: Hospital Anxiety and Depression Scale–Anxiety. In part (A): Model 1—the effect of pain intensity on SSAS. In part (B): Model 1—the effect of anxiety on coping strategies.

## 4. Discussion

The present study provides evidence that temporomandibular disorders (TMDs) are strongly influenced by psychological and somatosensory factors, supporting a biopsychosocial framework for understanding their clinical presentation. In the present sample, individuals with TMDs exhibited significantly higher levels of emotional distress and somatosensory amplification compared to healthy controls, a finding that aligns with previous research [33]. Earlier studies have consistently demonstrated that psychological factors such as anxiety, depression, somatization, and cognitive–emotional distress play a central role in the onset and persistence of TMDs symptoms [34, 35, 36]. Importantly, elevated somatosensory amplification scores may, in some individuals, reflect underlying traits related to health anxiety or somatic symptom tendencies, even in the absence of a formal psychiatric diagnosis. Such overlapping vulnerability factors may contribute to heightened bodily vigilance and symptom interpretation in TMDs and represent an important avenue for future research aimed at disentangling somatosensory amplification from broader somatic and health-related anxiety constructs.

Within the biopsychosocial model, coping strategies represent a key psychological mechanism linking emotional distress to pain perception and functional outcomes [37]. Coping refers to the cognitive and behavioral efforts individuals use to manage stressors, including chronic pain [38]. In chronic pain populations, greater reliance on emotion-focused or maladaptive (non-functional) coping strategies has been associated with higher pain intensity, increased emotional distress, and greater disability, whereas problem-focused coping is generally linked to better psychological adjustment and pain management [39]. Consistent with these theoretical assumptions, the present findings demonstrated that individuals with TMDs relied more heavily on emotion-focused and non-functional coping strategies, while problem-focused coping did not differ significantly from healthy controls. This pattern suggests that maladaptive coping may contribute to symptom persistence by amplifying pain-related vigilance, emotional distress, and somatosensory amplification in TMDs.

Gender emerged as an important factor influencing somatosensory amplification in the present study. Specifically, female gender was associated with higher levels of somatosensory amplification, indicating increased sensitivity to bodily sensations. Previous investigations have suggested that gender may exert a greater influence on temporomandibular dysfunction than dental or structural factors [40], with a higher prevalence of TMDs consistently reported among women [41]. Proposed explanations include lower pain thresholds and heightened pain sensitivity in women [42], as well as the combined influence of biological, hormonal, psychological, and social determinants [43]. Research has further suggested that increased joint laxity in females, potentially mediated by estrogen receptor activity on ligament metabolism, may increase vulnerability to TMDs symptoms [44]. The present findings extend this literature by demonstrating a direct association between female gender and somatosensory amplification. These findings underscore the clinical importance of sex-sensitive assessment, suggesting that female patients may particularly benefit from early identification and intervention targeting somatosensory amplification and maladaptive coping.

The present results also highlight the close interplay between pain intensity, emotional distress, somatosensory amplification, and coping strategies. Correlation and regression analyses demonstrated that higher pain intensity and emotional distress were significant predictors of somatosensory amplification, suggesting that physical and psychological factors act synergistically rather than independently in TMDs. These findings support previous evidence linking psychological distress [45], pain catastrophizing [46], fear-avoidance behaviors [47], and maladaptive coping patterns [48] to greater pain severity and disability in TMDs populations. Although TMD subtypes were not analyzed separately in this study due to sample size limitations, future research should explore whether different TMD subtypes exhibit distinct psychological patterns, which could further refine individualized assessment and intervention strategies.

From a clinical perspective, the identification of maladaptive coping strategies and emotional distress as predictors of somatosensory amplification highlights these factors as modifiable treatment targets. Psychological interventions such as cognitive-behavioral therapy (CBT), relaxation techniques, and biofeedback have been shown to improve coping flexibility, reduce emotional distress, and attenuate pain-related somatic sensitivity [49]. Furthermore, integrative treatment approaches that combine targeted coping interventions, structured psychological therapy, and anxiety management strategies could enhance TMD outcomes [36]. These approaches may include individualized CBT sessions focusing on pain-related thoughts, guided relaxation or mindfulness programs to reduce muscle tension and anxiety, and patient education on adaptive coping strategies to mitigate symptom persistence [50]. Implementing such interventions within routine clinical care may therefore improve both physical and psychological dimensions of TMD.

Moreover, depression and anxiety have been shown to influence masticatory muscle activity and coordination, potentially contributing to biomechanical strain, inflammatory responses, and secondary joint pain [51]. Abnormal trigeminal pain processing in TMDs, possibly related to serotonergic and catecholaminergic dysregulation, further supports the interaction between psychological and neurobiological mechanisms [52]. Given that the majority of participants in the present study were diagnosed with myogenous TMDs, the observed associations between pain intensity, emotional distress, coping strategies, and somatosensory amplification may be more strongly driven by muscle-related pain mechanisms rather than joint-specific pathology.

While some earlier studies suggested that psychological factors exert minimal influence on TMDs [53, 54], a substantial body of contemporary evidence contradicts this view, emphasizing the critical role of psychological and psychosocial variables in the onset, progression, and exacerbation of TMDs symptoms [55]. The present findings are consistent with this contemporary literature, reinforcing the importance of emotional distress, somatic sensitivity, and coping processes in shaping the clinical presentation of TMDs.

Overall, these results support the conceptualization of TMDs as a complex biopsychosocial condition rather than a purely structural or mechanical disorder. The interaction between nociceptive processing, emotional distress, somatosensory amplification, and coping behaviors underscores the need for multidimensional assessment and integrative treatment approaches that address both physical and psychological components of the disorder.

## 5. Conclusions

In conclusion, this study highlights the associations between TMDs pain severity, psychological symptoms, and coping strategies. Higher pain intensity and longer duration of TMDs were linked to increased psychological distress and greater reliance on maladaptive coping methods. These findings underscore the importance of adopting integrated TMD assessment protocols that systematically include psychological screening alongside routine physical evaluation.

Screening for anxiety and coping strategies in dental practice can help identify patients at risk of heightened pain sensitivity or maladaptive coping, enabling timely and targeted interventions. Early identification of emotional distress heightened somatosensory amplification, and maladaptive coping patterns may allow for timely psychological and behavioral interventions. Such early, integrated approaches have the potential to reduce symptom persistence, limit the transition from acute to chronic TMDs, and ultimately improve long-term clinical outcomes. Incorporating biopsychosocial screening and early intervention strategies into standard TMD care may therefore represent a critical step toward more effective and sustainable management of this complex condition. Overall, the findings of this study may contribute to guiding more individualized and integrated treatment pathways for patients with TMDs by supporting timely referral to multidisciplinary care, including psychological and behavioral interventions, alongside conventional dental management.

## 6. Limitations

This study has several limitations. First, its cross-sectional design precludes causal inferences among pain intensity, psychological factors, coping strategies, and somatosensory amplification. Second, reliance on self-report instruments may have introduced recall and social desirability bias, particularly in the assessment of coping strategies using the COPE inventory. Third, the sample was drawn from a single center with a relatively limited size, which may restrict generalizability. In addition, objective physiological or imaging measures (*e.g.*, Electromyography (EMG) and magnetic resonance imaging (MRI)) were not included, and relevant psychosocial constructs such as somatization and stress were not directly assessed. Furthermore, socio-economic status, education level, and occupational stress factors that may influence anxiety and coping patterns were not systematically controlled for in the present study and should be considered when interpreting the findings. Although depression frequently co-occurs with anxiety in TMD populations, it was not included in the present analysis in order to maintain a focused analytical framework and to reduce construct overlap, as anxiety has been shown to be more directly associated with pain-related vigilance and somatosensory amplification. TMD subtypes were also not analyzed separately due to sample size constraints. Future longitudinal and multimodal studies are warranted.
